# Multi-resolution speech analysis for automatic speech recognition using deep neural networks: Experiments on TIMIT

**DOI:** 10.1371/journal.pone.0205355

**Published:** 2018-10-10

**Authors:** Doroteo T. Toledano, María Pilar Fernández-Gallego, Alicia Lozano-Diez

**Affiliations:** AuDIaS - Audio, Data Intelligence and Speech, Universidad Autónoma de Madrid, Madrid, Spain; Universite Paris Diderot/CNRS, UNITED STATES

## Abstract

Speech Analysis for Automatic Speech Recognition (ASR) systems typically starts with a Short-Time Fourier Transform (STFT) that implies selecting a fixed point in the time-frequency resolution trade-off. This approach, combined with a Mel-frequency scaled filterbank and a Discrete Cosine Transform give rise to the Mel-Frequency Cepstral Coefficients (MFCC), which have been the most common speech features in speech processing for the last decades. These features were particularly well suited for the previous Hidden Markov Models/Gaussian Mixture Models (HMM/GMM) state of the art in ASR. In particular they produced highly uncorrelated features of small dimensionality (typically 13 coefficients plus deltas and double deltas), which was very convenient for diagonal covariance GMMs, for dealing with the curse of dimensionality and for the limited computing resources of a decade ago. Currently most ASR systems use Deep Neural Networks (DNN) instead of the GMMs for modeling the acoustic features, which provides more flexibility regarding the definition of the features. In particular, acoustic features can be highly correlated and can be much larger in size because the DNNs are very powerful at processing high-dimensionality inputs. Also, the computing hardware has reached a level of evolution that makes computational cost in speech processing a less relevant issue. In this context we have decided to revisit the problem of the time-frequency resolution in speech analysis, and in particular to check if multi-resolution speech analysis (both in time and frequency) can be helpful in improving acoustic modeling using DNNs. Our experiments start with several Kaldi baseline system for the well known TIMIT corpus and modify them by adding multi-resolution speech representations by concatenating different spectra computed using different time-frequency resolutions and different post-processed and speaker-adapted features using different time-frequency resolutions. Our experiments show that using a multi-resolution speech representation tends to improve over results using the baseline single resolution speech representation, which seems to confirm our main hypothesis. However, results combining multi-resolution with the highly post-processed and speaker-adapted features, which provide the best results in Kaldi for TIMIT, yield only very modest improvements.

## Introduction

Automatic speech recognition (ASR) aims at converting speech signals into textual representations and is an essential part in data analysis applications that process multimedia (audio/video) content, such as keyword spotting and speaker detection, and in applications that use voice in human-machine interfaces, such as intelligent personal assistants, interactive voice response (IVR) systems and voice search, to name a few.

For a period of over two decades, the main paradigm in ASR was to use Hidden Markov Models (HMMs) to model the temporal evolution of speech and Gaussian Mixture Models (GMMs) to model the acoustic characteristics of speech at each phonetic state [1], using statistical *n-gram* language models to improve recognition accuracy by modeling the probabilities of different word sequences. In the last few years, ASR has experienced a rapid improvement in accuracy, mainly driven by the adoption of the recent advances in deep learning [2]. Deep learning, and in particular deep neural networks (DNNs), have replaced GMMs to model the acoustic characteristics of speech at each phonetic state, but the rest of the architecture is still kept for many practical systems. This gives rise to what is usually called *hybrid* ASR systems, because they make use of the classic HMM/GMM architecture and, only after the HMM/GMM system has been trained, replace the GMM with a DNN whose role is to estimate the posterior probabilities of each HMM state, given the acoustic input. Recurrent Neural Networks (RNNs) have also been used successfully for language modeling as a replacement for the classical statistical n-gram models [3] [4].

There is currently active research in developing what is called *end-to-end* ASR approaches in which all the *old* HMM/GMM machinery is dropped and DNNs are directly trained just from speech and word/phone transcriptions to optimize a *loss function* related to the ASR problem, such as the Word or Phone Error Rates (WER, PER). This approach has the theoretical advantage that the optimization is performed in a single step and with the direct goal of improving ASR accuracy, whereas in the *hybrid* approach the DNNs are trained to minimize the cross-entropy between the predicted and actual HMM states, which is obviously related, but not directly the ASR accuracy. The *end-to-end* approach, on the other hand, requires the DNNs to handle the time evolution of the speech signal, which leads naturally to the use of Recurrent Neural Networks (RNNs) such as Long Short-Term Memory (LSTM) RNNs [5] and more recently Gated Recurrent Unit (GRU) RNNs [6]. The target task in the *end-to-end* ASR approach is a sequence-to-sequence mapping in which a sequence of feature vectors, typically obtained from the speech signal each 10 ms., has to be mapped to a (much shorter) sequence of phones or words, and the loss to optimize is the phone/word error rate.

Probably the first success in these attempts was the introduction of the Connectionist Temporal Classification (CTC) approach [7], which achieved excellent results on the TIMIT corpus [8]. TIMIT is very commonly used in the ASR community to compare acoustic-phonetic modeling, particularly at early stages of a novel proposal, but it is small and does not contain the usual problems found in realistic ASR such as background noise and spontaneous speech. CTC was later successfully applied to more complex speech recognition corpora such as the Wall Street Journal corpus [9] and the Switchboard corpus [10].

More recently, attention models have been introduced in deep learning systems to help DNNs focus their attention on specific parts of the input (for instance in relevant parts of an image [11]), and have also been applied to help RNNs focus their attention on specific parts of a speech signal to allow *end-to-end* DNN speech recognition with excellent results [12] on TIMIT and also in a larger corpus such as the Wall Street Journal corpus [13].

Although the search for new deep learning paradigms to improve ASR is very exciting, we have decided to focus our research on a different area. In particular, in this article we are interested in trying to find representations of the speech signal that can improve ASR using different deep learning approaches, and explore the use of a multi-resolution (both in time and in frequency) representation of the speech signal to facilitate DNNs learning of the acoustic characteristics of the different phones.

### Motivation

There are a number of reasons to start this research at this point. First, the acoustic-phonetic models employed in ASR have changed from Gaussian Mixture Models (GMMs) to Deep Neural Networks (DNNs). This means that the best speech representation (parametrization) developed for GMMs (Mel-Frequency Cepstral Coefficients, MFCCs, usually with some post-processing) may well not be the best possible representation for DNNs. For instance, GMMs in ASR were typically modeled using a diagonal covariance matrix, which means that the different dimensions of the feature vector are assumed to be uncorrelated. This is approximately true for MFCCs thanks to the Discrete Cosine Transform (DCT) that is applied to the Mel-frequency scaled filterbank outputs, achieving a high degree of uncorrelation among MFCCs. These filterbank outputs are themselves strongly correlated and therefore they are a bad choice to be modeled with a diagonal covariance GMM. On the other hand, DNNs do not require their inputs to be uncorrelated, and therefore can deal very well with the outputs of the filterbank, achieving even better results than using MFCCs [2].

A perhaps stronger difference is that deep learning came with the promise of its capability to deal with raw signals without the need for hand-crafted features [14]. The rationale is that the successive levels of information processing corresponding to the different layers of the DNNs are able to extract successively more complex features. While for image processing it is quite common to have the raw image as input to the DNN, in the case of speech processing it is still much more common to use some form of speech processing and use as input for the DNNs the output of a mel-scaled filterbank or even the classic MFCCs [2]. Typical dimensionalities per 10 ms. speech frame considering the different speech representations are: 13 MFCCs, 20-40 filterbank outputs, 129 or 257 spectral coefficients, or 200 / 400 waveform samples for a 25 ms. window and 8 / 16 KHz. sampling. This leads to another important difference between GMMs and DNNs: while GMMs are heavily affected by *the curse of dimensionality*, it is well known [15] that Neural Networks can cope very well with high dimensionality inputs. For this reason features used in the HMM/GMM paradigm need to be very compact, while for DNNs the impact of input dimensionality is mainly in computational complexity, a factor that the technological evolution of hardware tends to make less relevant. For this reason, we will not try to avoid high dimensionality in the input, which will be required for multi-resolution representations of the speech signal.

One of the main motivations for using a multi-resolution representation of the speech signal arises from the different duration of the phones in speech. Table 1 presents duration statistics for the longest and shortest phones in the TIMIT corpus (considering the reduction from the original 60 phones to the set of 48 phones proposed in [16] for acoustic modeling). It can be observed that the mean duration ranges from less than 18 ms. (for the /*b*/ phone) to over 150 ms. (for the /*aw*/ phone). In the HMM/GMM setup it was customary to use a speech analysis stage based on windows (typically Hamming) with a length of 25 ms. and advancing 10 ms. from one window to the next. This was found to be a good approximation on average (in fact it is smaller than the average duration of most phones) but it is possible that using longer and shorter windows could be beneficial for certain phones. In the HMM/GMM setup this possibility was impractical due to the negative effects of the curse of dimensionality as the feature input vectors increased with multi-resolution representations of speech, but this should not be an important problem with the advent of DNNs for acoustic modeling.

**Table 1:** TIMIT phone duration statistics for the longest (/*aw*/) and shortest (/*b*/) phones in the set of 48 phones used for training. Columns show mean, standard deviation, maximum and minimum duration. The last column indicates the percentage of phones with a duration shorter than 25ms.

Phone	Mean (ms)	SD (ms)	Max (ms)	Min (ms)	< 25ms (%)
/*aw*/	163.62	51.59	415.00	62.69	0.00
/*b*/	17.50	7.16	98.63	2.94	86.08

**Fig 1:**
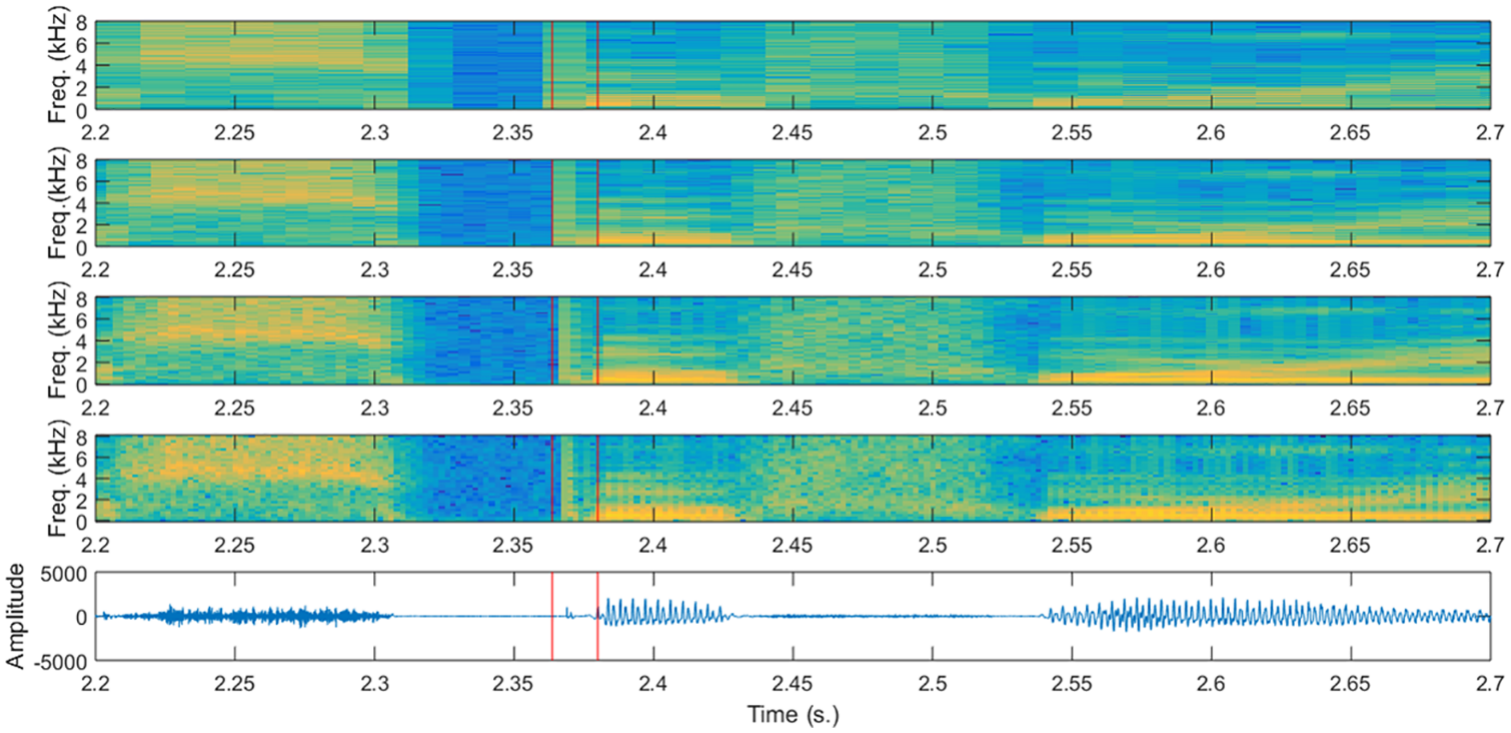
Spectrograms with different time-resolution trade-offs for a short phone. Spectrograms obtained for a segment of 0.5 seconds around the /*b*/ phone in the word *before* in the sentence “Drop five forms in the box before you go out” (Speaker FAKS0, sentence SX313). The only difference in the spectrograms is the length of the Hamming windows used: 32, 16, 8 and 4 ms. from top to bottom. Vertical red lines show the limits of the /*b*/ phone. There are substantial differences in the spectral representation of the short /*b*/ phone, for which an analysis using shorter windows is probably better.

**Fig 2:**
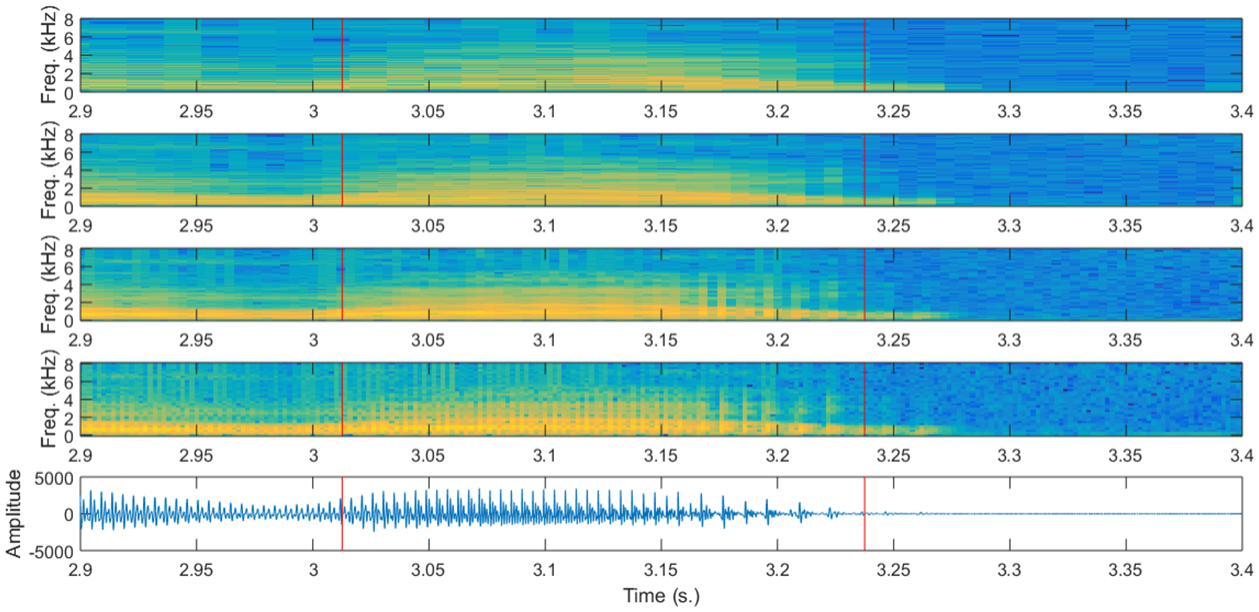
Spectrograms with different time-resolution trade-offs for a long phone. Spectrograms obtained for a segment of 0.5 seconds around the /*aw*/ phone (more precisely, dipthong) of “out” in the sentence “Drop five forms in the box before you go out” (Speaker FAKS0, sentence SX313). The only difference in the spectrograms is the length of the Hamming windows used: 32, 16, 8 and 4 ms. from top to bottom. Vertical red lines show the limits of the /*aw*/ dipthong. There are substantial differences in the spectral representation of the long /*aw*/ dipthong, for which an analysis using longer windows is probably better.

Having all that in mind, this article tries to determine whether acoustic modeling can benefit from using multi-resolution spectral representations of speech. Figs 1 and 2 represent several spectral representations of two segments of the same phrase (“Drop five forms in the box before you go out”) which contains two examples of the shortest phone (/*b*/) and an example of the longest phone (/*aw*/). It can be seen that, particularly for the shortest phones there is a big difference in the spectrogram obtained with different windows. Our hope is that having multiple spectral representations can be beneficial for acoustic modeling with DNNs, particularly for these short phones, but maybe also for longer phones.

### Paper organization

The rest of the paper is organized as follows. First, Section Speech analysis and time-frequency resolution provides a brief introduction to speech analysis and the limitations in time and frequency. DNNs and their use in speech processing are introduced in Section Deep neural networks and speech processing. After that, we will present the dataset, tools and evaluation metrics in Section Materials and methods. Then, the baseline systems will be described in Section Baseline systems. In Section Multi-resolution systems we will describe the systems we have developed to analyze if a multi-resolution representation of the speech signal can help in acoustic modeling with DNNs. After that, experimental results will be presented and analyzed in Section Results and discussion, and finally conclusions will be presented in Section Conclusion.

## Speech analysis and time-frequency resolution

### Short Time Fourier Transform (STFT) and time-frequency resolution

The analysis of the speech signal typically starts with a time-dependent Fourier transform, also called *Short-Time Fourier Transform* (STFT), which is a method to analyze signals whose Fourier transform (i.e. *spectrum*) changes over time, as it is the case of the speech signal. The STFT of a signal *x*[*n*] is defined as

$$\begin{array}{c} X\left[n,\omega \right)=\sum_{m=-\infty }^{\infty }x\left[n+m\right]w\left[m\right]e^{-j\omega m}=\text{DTFT}\left\{x[n+m]w[m]\right\} \end{array}$$ (1)

were *w*[*m*] is a *window* signal, *n* is the sample of *x*[*m*] at which we start applying the window (i.e. the time at which our analysis starts) and *ω* is the discrete-time frequency in rads/sample. The STFT is in fact the Discrete-Time Fourier Transform (DTFT) of the time-displaced signal *x*[*n* + *m*] multiplied by the window *w*[*m*] where the DTFT is given by

$$\begin{array}{c} \text{DTFT}\left\{x[m]\right\}=X\left(\omega \right)=\sum_{m=-\infty }^{\infty }x\left[m\right]e^{-j\omega m} \end{array}$$ (2)

The DTFT is defined on a continuous frequency variable *ω* which makes it not computable in finite time. However, for finite-duration signals constrained to be null outside [0, *N* − 1], samples of the DTFT at *ω* = 2*πk*/*N* can be computed using the Discrete Fourier Transform (DFT)

$$\begin{array}{c} {\text{DFT}}_{N}\left\{x[m]\right\}=X\left[k\right]=X\left(\omega_{k}=\frac{2\pi k}{N}\right)=\sum_{m=0}^{N-1}x\left[m\right]e^{-j\omega m} \end{array}$$ (3)

which can be computed efficiently using the Fast Fourier Transform (FFT) algorithm.

If we choose a window *w*[*m*] constrained to be null outside [0, *L* − 1] with *L* ≤ *N*, we can replace the DTFT by the DFT in the definition of the STFT to get to the STFT sampled in frequency

$$\begin{array}{c} X\left[n,k\right]=X\left[n,\omega_{k}=\frac{2\pi k}{N}\right)=\sum_{m=0}^{N-1}x\left[n+m\right]w\left[m\right]e^{-j\frac{2\pi km}{N}}={\text{DFT}}_{N}\left\{x[n+m]w[m]\right\} \end{array}$$ (4)

While the STFT sampled in frequency can be computed in finite time, in practice computing the whole STFT sampled in frequency would be a waste of time because it computes a spectrum per input signal sample, and two consecutive spectra would differ very little since the time signals from which they are computed differ only in one sample. To get a spectral representation of a time-varying signal more efficiently, the STFT is also sampled in time each *R* samples, which gives rise to the STFT sampled in time and frequency

$$\begin{array}{c} X_{r}\left[k\right]=X\left[rR,k\right]=X\left[rR,\omega_{k}=\frac{2\pi k}{N}\right)={\text{DFT}}_{N}\left\{x[rR+m]w[m]\right\} \end{array}$$ (5)

In this way, a spectrum is computed every *R* samples of the input signal (a frame period, where *r* is the frame number) and as long as *R*≤*L* (assuming also *L*≤*N*) the STFT is invertible (i.e. no information is lost in the transformation).

A typical setting for speech analysis is to use windows *w*[*m*] of length 25 ms. and a displacement between spectra computations of 10 ms. For a signal *x*[*m*] sampled at 16000 Hz. this means selecting *R* = 160 and *L* = 400 samples, for which normally a DFT of *N* = 512 points is used.

While this analysis of the speech signal does not lose information, it implies selecting a particular point in the trade-off between time and frequency resolution.

It is obvious that the time resolution is affected by the time sampling of the STFT because a spectrum is computed each *R* samples of the input signal, and therefore events of the signal less than *R* samples apart could be mixed in the speech analysis. This means that with the typical speech analysis used it would be difficult to distinguish events closer than 10 ms. However, the time resolution is also affected by the window length *L* since the window restricts the amount of signal that is considered to compute a single spectrum (DFT). Computing a DFT removes the time variable, and therefore it means that the spectrum will depend on the signal observed through that window. The effect of having longer windows is that short events tend to extend their apparent effect in the spectrogram for at least the duration of the window, as it can be observed in Fig 1, which means that it could be difficult to distinguish events closer than 25 ms. for a typical setting in speech analysis for ASR.

The frequency resolution is affected by the sampling in frequency produced by using the DTF instead of the DTFT. This sampling is produced at *ω*_*k*_ = 2*πk*/*N* in rads/s, or equivalently at *f*_*k*_ = *k*/*NT*_*S*_ Hz., where *T*_*S*_ = 1/*f*_*S*_ is the sampling period in seconds. For a typical setting of *N* = 512 (or *N* = 256) and a sampling frequency of 16000 Hz. we obtain one sample of the spectrum every 31.25 Hz. (or 62.5 Hz.). However, more important than this effect is the reduction of the frequency resolution imposed by windowing. When a signal is multiplied by a window, its spectrum is convolved with the spectrum of the window as stated by the multiplication property of the DTFT

$$\begin{array}{c} x\left[n\right]w\left[n\right]\;\underset{↔}{\text{DTFT}}\frac{1}{2\pi }\int_{0}^{2\pi }X\left(e^{j\theta }\right)W\left(e^{j\left(\omega -\theta \right)}\right)d\theta \end{array}$$ (6)

As a consequence, the spectrum of the windowed signal is *blurred* or *smoothed* by the effect of the main lobe of the spectrum of the window. As a consequence the frequency resolution of the STFT analysis is also reduced by this effect, which makes difficult to distinguish frequencies that are closer than the width of the main lobe of the window spectrum. The main lobe of a window is approximately inversely proportional to the length of the window, *L*, so for instance for the Hamming window the width of the main lobe is 8*π*/*L* − 1 rads/s [17]. For the typical case in speech processing of windows of 25 ms. (which means *L* = 400 samples for *f*_*S*_ = 16000 Hz.) the width of the main lobe is 8*π*/399 rads/s or 4*f*_*S*_/399 = 160.4 Hz. The effect of windowing on frequency resolution is normally more important than the effect of sampling in frequency of the STFT analysis as long as *N* ≥ *L*.

Given the previous analysis, we can conclude that a typical STFT analysis used in speech processing has a time resolution of about 25 ms. and a frequency resolution of about 160 Hz. (being both conservative estimations). In this work, we try to explore if using more than just one time-frequency resolution in the speech analysis can help DNNs for ASR acoustic modeling. In order to do so, we need to modify some of the parameters of the STFT analysis:

- To improve time resolution we need to reduce *R* and *L*. However, reducing *L* will also worsen frequency resolution.
- To improve frequency resolution we need to increase *L* (we have decided not to experiment with different window types at the moment), which will also worsen time resolution.

To use different time-frequency resolutions we will experiment with several STFTs using different values of *R* and *L* and make DNNs have as input several STFT representations of the signal (or more complex features derived from them). Instead of Fourier analysis, we could have used Wavelets, which are a direct way to represent signals at different time-frequency scales, but using STFT at this point made comparisons easier.

### Filterbank analysis, MFCC and frequency resolution

The STFT is just the first part of speech analysis, but normally DNNs do not take as input the spectrum directly. Instead, they usually take more compact features derived from the results of the STFT. Here we will briefly review the most common modifications giving rise to the Mel-frequency cepstral coefficients (MFCC), paying particular attention to the influence of these transformations in the time-frequency resolution.

The phase of the STFT is discarded in speech analysis and only the module (|*X*_*r*_[*k*]|) is kept, based on the knowledge that the human auditory system is somewhat insensitive to the phase information. This operation does not affect the time-frequency resolution.

To model the non-uniform frequency response of the human auditory system a filterbank using a non-uniform frequency transformation is applied to this power spectral density. One of the most common non-uniform frequency transformations is the Mel frequency scale [18]:

$$\begin{array}{c} Mel\left(f\right)=2596log_{10}(1+\frac{f}{700}) \end{array}$$ (7)

**Fig 3:**
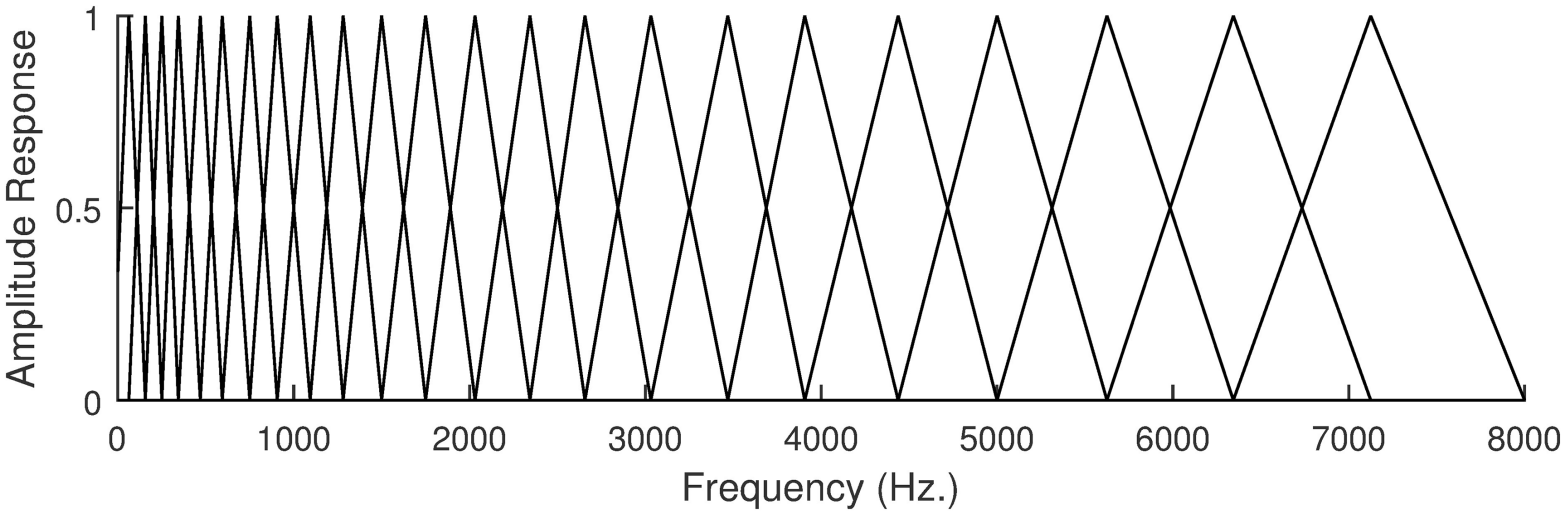
Mel-frequency scaled filterbank. The figure shows the amplitude response of the 23 filters of a Mel-scaled filterbank ranging from 0 to 8000 Hz.

Typically, between *M* = 20 and *M* = 40 triangular filters are evenly distributed in the Mel-frequency scale (see Fig 3). The output of each of these filters for |*X*_*r*_[*k*]| in logarithmic scale (dB) constitutes the filterbank features, *FB*_*r*_[*j*] where *j* now is the index of the filter. Clearly the filterbank analysis affects the frequency (but not the time) resolution, providing less frequency resolution for a smaller number of filters.

Finally, a Discrete Cosine Transform (DCT) is applied to the filterbank features,

$$\begin{array}{c} C_{r}\left[i\right]=\sqrt[{}]{\frac{2}{M}}\sum_{j=0}^{M}FB_{r}\left[j\right]cos\;(\frac{\pi i}{M}(j-0.5)) \end{array}$$ (8)

keeping typically the first *C* = 13 DCT coefficients as the final MFCC features. The main effect of keeping less DCT coefficients than the number of filterbank features is *smoothing* the filterbank output in a process that loses some information. This means that decreasing the number of coefficients kept we reduce the frequency resolution again. If *M* = *C* (i.e. we use the same number of MFCC coefficients as filters in the filterbank) the DCT is invertible and therefore no information is lost and therefore the frequency resolution remains the same.

After this analysis we can conclude that the operations to obtain the filterbank features or the MFCC features from the STFT affect the frequency resolution of the analysis but not the time resolution. Therefore we will have to take into account the number of filters in the filterbank analysis, *M*, and the number of MFCC features computed, *C*, in our experiments.

## Deep neural networks and speech processing

**Fig 4:**
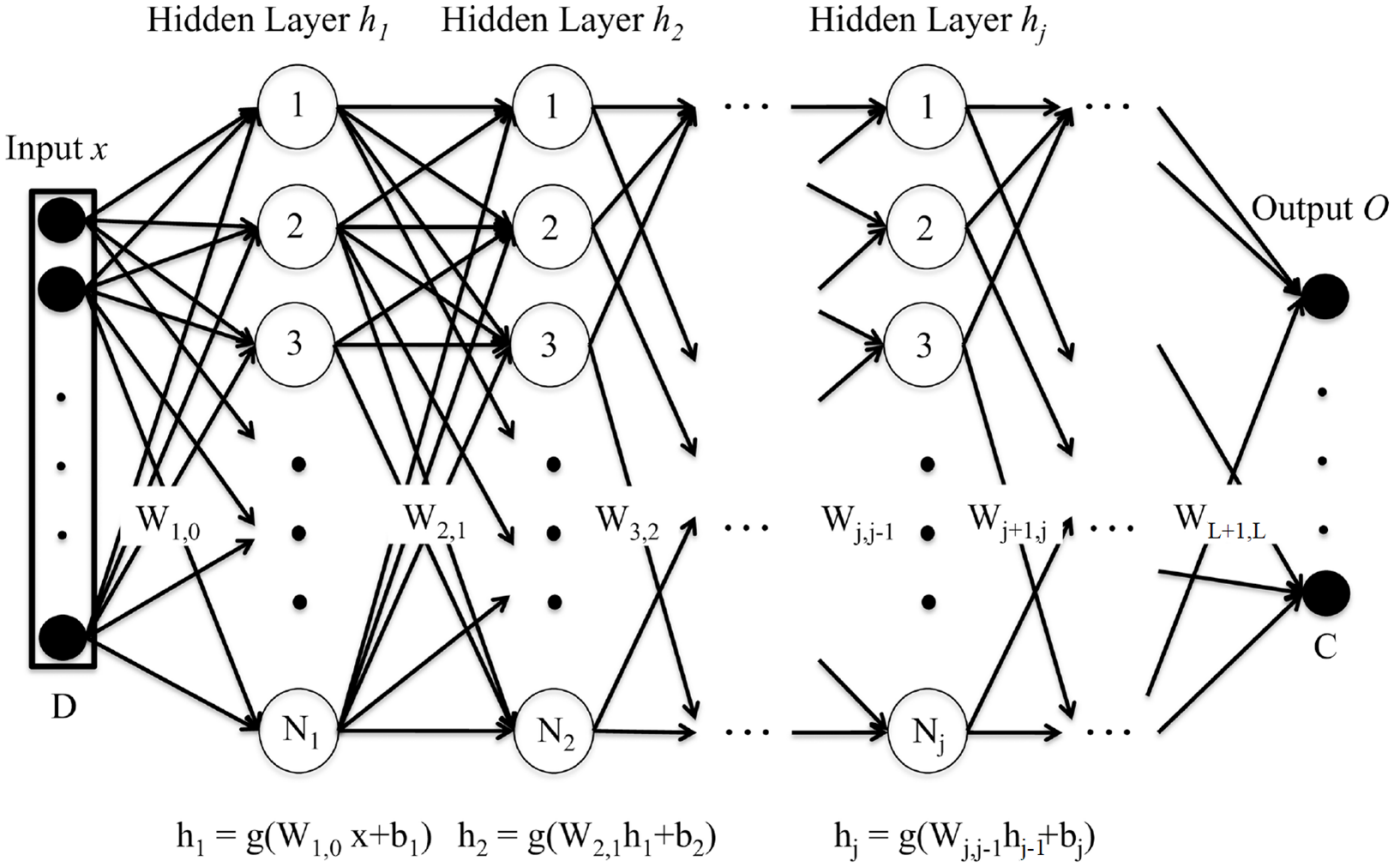
Deep Neural Network (DNN). This is a graphical representation of a standard feedforward DNN architecture. The DNN is fed with an input vector *x* of dimension D which is transformed by the hidden layers *h*_*j*_ (composed of *N*_*j*_ hidden units) according to an activation function *g* and the parameters of the DNN (weight matrices *W* and bias vectors *b*). Finally the output layer *O* produces the output of the DNN for the target task (for the case of classification, the posterior probability of an input vector to belong to each of the C classes). Reprinted from [19] under a CC BY license, with permission from Alicia Lozano et. al., original copyright 2017.

Deep neural networks (DNN) are machine learning tools which allow for the learning of complex non-linear multidimensional functions of a given input in order to minimize an error cost. A graphical example of a standard deep neural network is presented in Fig 4.

This way, a feedforward DNN used to perform a classification task might have the following general structure: an input layer, which is fed with some input vectors representing the data; two or more hidden layers (in opposition to shallow architectures, which had just one hidden layer), where a transformation is applied to the output of the previous layer, obtaining a higher level representation as we move away from the input layer; and an output layer, which computes the output of the DNN.

The model is defined by its parameters: weight matrices, *W*_*j*,*j*−1_, and bias vectors, *b*_*j*_, with *j* going from 1 to the number of hidden layers plus one. Given an input vector *x*, each hidden layer applies a non-linear activation function *g* to an affine transformation of the output of the previous layer defined by the weight matrices, *W*_*j*,*j*−1_, and bias vectors, *b*_*j*_, according to the following equations:

$$\begin{array}{c} h_{1}\left(x\right)=g(W_{1,0}x+b_{1}) \end{array}$$ (9)

$$\begin{array}{c} h_{j}\left(x\right)=g(W_{j,j-1}h_{j-1}\left(x\right)+b_{j}),\;\;j=2,\ldots ,N-1 \end{array}$$ (10)

In these equations the non-linear activation function of the hidden layers, *g*, is often *sigmoid*, hyperbolic tangent (*tanh*) or Rectified Linear Unit (ReLU) or some of its variants. For a classification task, the output layer uses normally a *softmax* activation function that normalizes the outputs to be in the interval [0, 1] and sum to one, so that they can be interpreted, for a well trained network, as the posterior probabilities of the different classes *c*, given the input *x*:

$$\begin{array}{c} P\left(c|x\right)=g_{\text{softmax}}(W_{L}^{c}h_{L}\left(x\right)+b_{c})=\frac{\text{exp}(W_{L}^{c}h_{L}\left(x\right)+b_{c})}{\sum_{c=1}^{C}\text{exp}(W_{L}^{c}h_{L}\left(x\right)+b_{c})} \end{array}$$ (11)

where *h*_*L*_(*x*) refers to the last hidden layer activations for input *x*, $W_{L}^{c}$ is the weight vector connecting the last hidden layer and the output unit for class *c*, *b*_*c*_ is the bias value of the output unit for class *c* and there are a total of *C* possible classes.

For a classification task, supervised training starts with a training set (*x*_*i*_, *y*_*i*_) where *x*_*i*_ is a given input vector and *y*_*i*_ a vector representing the output for the true (or target) class (for classification tasks, normally a one-hot codification of the output is used, in which a 1 is assigned to the correct class and 0 are assigned to the rest). The parameters to adjust in the learning process are the weight matrices and the bias vectors. These are normally initialized in a random way and then adjusted iteratively to minimize the cost function, typically with backpropagation and stochastic gradient descent or other optimization approaches. For this purpose, in the last layer, the output is compared to the reference label (true or target value) and the errors are back-propagated to modify the weights. In the early stages of deep learning, and particularly for limited training sets, it was found useful employing unsupervised initialization of the DNN weights using Restricted Boltzmann Machines (RBMs) to find suitable weights and biases in a layer-by-layer way [20]. With large datasets and other strategies nowadays this is not common in practice.

## Materials and methods

This section describes the dataset, the evaluation metrics and the tools used for the experimental part of this research paper.

### Dataset

The experiments presented in this paper have been carried out on the well-known TIMIT corpus [21]. This corpus contains read speech spoken by a total of 630 speakers covering 8 dialectal regions in the U.S. Each speaker read the same 2 dialectal sentences (SA sentences) designed to expose dialectal differences, 5 out of 450 phonetically balanced sentences (SX sentences) and 3 out of 1890 phonetically diverse sentences (SI sentences). This provides a total of 6300 utterances. The 30% of speakers were female, while the remaining 70% were male. The audio is distributed with a sampling frequency of 16 KHz. and a signed, linear, 16 bits per sample encoding.

The corpus defines a *core test set* composed of 24 speakers but excluding the SA sentences, which yields a total of 192 utterances. It also defines a *complete test set* that includes the *core test test* plus all the utterances spoken by speakers in the *core test set*. This complete test set includes 168 speakers and 1344 utterances (again the SA sentences are excluded). Finally, the corpus defines a *train set* that includes the utterances read by the remaining 462 speakers.

### Evaluation metrics

In all the experiments the SA sentences were excluded both for training and testing, as it is common practice. Also the original set of 60 phones is mapped to a reduced set of 48 phones for training the acoustic models, and these are mapped to an even more reduced set of 39 phones for Phone Error Rate (PER) measurement, as is customary since the paper by Lee and Hon [16]. All the speakers in the *train set* were used for training. The main results are provided on the *core test set*, while we also provide results on a *development set* composed of 50 speakers from the *test set* that are not included in the *core test set*. All results include a phone bigram language model trained on the training set.

The primary evaluation metric is the Phone Error Rate (PER) computed with a reduced set of 39 phones as in [16], and defined as

$$\begin{array}{c} PER=\frac{S+I+D}{N}\times 100\;\;\left(\%\right) \end{array}$$ (12)

were *S*, *I* and *D* are the number of phone substitutions, insertions and deletions found when comparing the recognized and the reference phone transcriptions, and *N* is the total number of reference (true) phones in the evaluation set.

In some experiments we also use the frame-by-frame phone state classification accuracy, expressed as the percentage of frames in the evaluation set for which the phone state predicted by the DNN coincides with the phone state determined in a forced alignment performed with a previously trained HMM/GMM system.

### Tools

For the experiments we have used one of the most commonly used software packages in speech recognition research: Kaldi [22]. We have also used HTK [18], mainly to analyze the results obtained with Kaldi in more detail. We have also performed a set of experiments, focusing on the frame classification accuracy of the DNNs and not in the overall speech recognition performance using standard deep learning packages such as Theano [23] and KERAS [24]. The spectrogram features were computed using MATLAB [25].

## Baseline systems

The baseline systems chosen for our experiments are the standard Kaldi DNN recipes for TIMIT included in the Kaldi distribution. In all cases (both for baseline systems and the systems proposed in this article) the recipe starts with a common training procedure for the HMM/GMM system, which includes:

- **STFT:** Computation of the STFT using windows of size 25 ms. (*L* = 400 for *f*_*S*_ = 16000 Hz.) and a frame shift of 10 ms. (*R* = 160 for *f*_*S*_ = 16000 Hz.) with FFTs of *N* = 512 points. The window used in the STFT is a special window called *povey*, named after the main developer of Kaldi, but results using the Hamming window are similar.
- **MFCC:** Computation of 13 MFCCs per frame using a Mel-scaled filterbank with 23 triangular filters distributed between 20 Hz. and 8000 Hz.
- **CMVN:** Cepstral Mean and Variance Normalization. It consists in normalizing the mean and variance of the MFCCs to make them have zero mean and unit variance. The baseline Kaldi recipes applies CMVN per speaker.
- **Monophones:** Training of a monophone HMM/GMM system starting from an uniform segmentation of the files and iteratively re-aligning the data.
- **Triphones1:** Training of a triphone HMM/GMM system using MFCC + delta + delta-delta features.
- **Triphones2:** Training a more advanced triphone HMM/GMM system using features derived from the MFCCs. In particular the MFCC features are spliced taking the central frame ±4 frames and these matrices are projected into vectors of 40 dimensions using LDA. Later these vectors are diagonalized using Maximum Likelihood Linear Transform (MLLT) [26].
- **Triphones3**: Finally, Speaker Adapted Training (SAT) is performed. This consists in applying a feature-domain speaker adaptation technique known as feature-space Maximum Likelihood Linear Regression (fMLLR) [27] that transforms the features and training the triphone HMM/GMM system using these speaker-adapted features.

These first steps for training the HMM/GMM system are shared across multiple recipes for many different databases, and are a very common way to start training the acoustic models. All the *hybrid* systems in this article are built from these HMM/GMM systems.

For the experiments in this article we have considered two baseline *hybrid* systems included in the standard Kaldi distribution:

- **FC-sigmoid-RBM-pretrain:** The first one (developed by Karel Vesely and corresponding to the *nnet1* setup in Kaldi, http://kaldi-asr.org/doc/dnn1.html) uses a Fully Connected (FC) DNN with six hidden layers of 1024 units each, with *sigmoid* activation function, plus an output layer with 1936 outputs and *softmax* activation funcion. In this case, the DNN is pre-trained layer by layer in an unsupervised way using Restricted Boltzmann Machines (RBMs) [20]. Later the weights and biases are trained using Stochastic Gradient Descent and cross-entropy, using a validation set (10% of the training set reserved for this purpose only) for early stopping. Typically training stops after about 13 epochs. The input for the DNN are the features used for **Triphones3** spliced to include the central frame ±5 frames. The phone state alignments used are those obtained using the **Triphones3** models.
- **FC-tanh:** The second one (developed by Daniel Povey and corresponding to the *nnet2* setup in Kaldi, http://kaldi-asr.org/doc/dnn2.html) uses a Fully Connected (FC) DNN with two hidden layers of 300 units and *tanh* activation functions, and an output output layer of 1947 units and *softmax* activation function. Its weights are initialized randomly and trained for a total of 30 iterations of 200K examples using a parallel approximation of Natural Gradient for Stochastic Gradient Descent [28] and the cross-entropy as loss function. The network is initially trained with a single hidden layer and the second hidden layer is added after two iterations. The output units are augmented after 12 iterations to 3136 *mixtures* (i.e. the *softmax* layer with 1947 units is augmented to a larger *softmax* layer and a last layer is added that sums the *softmax* outputs corresponding to the same original output unit). There are also many details that we cannot cover in this article, such as preconditioning in the hidden layers and a decorrelation input transformation, which are described in the Kaldi *nnet2* setup documentation. The inputs for the DNN are basically the same features used for **Triphones3** with a ±4 frame splicing so that the input dimension is 360. The phone state alignments used as targets are those obtained using the **Triphones3** models.

Apart from these baseline systems included in the Kaldi recipes for TIMIT, we have built other two baseline systems based on Kaldi recipes for another corpora:

- **FC-pnorm:** This network (developed by Daniel Povey and also corresponding to the *nnet2* setup in Kaldi, http://kaldi-asr.org/doc/dnn2.html) is similar to the **FC-tanh** network. The main difference is that instead of using the *tanh* activation function it uses the *p* − *norm* activation functions [29], which are dimensionality reducing non-linearities inspired in *maxout*, but outputting for a group of *G* units the *p* − *norm* value of the *G*-dimensional vector instead of just the maximum:
  $$\begin{array}{c} y={\left||x|\right|}_{p}={\left(\sum_{i=1}^{G}|x_{i}{|}^{p}\right)}^{1/p} \end{array}$$ (13)
  where the sum is over the set of input units that are grouped together. In the particular setup used here *p* = 2 and the group size is *G* = 10. The two hidden layers have 3000 units, which are then reduced to 300 units with the *p* − *norm* non linearity. Additionally, the outputs of the *p* − *norm* non-linearities are normalized to avoid the outputs grow over a RMS value of 1 to facilitate convergence. Another difference is that the last layer is not augmented as in the **FC-tanh** network. The rest of the network is similar to the **FC-tanh** network.
- **TDNN-pnorm** This network (developed by Daniel Povey and corresponding to the *nnet3* setup in Kaldi, http://kaldi-asr.org/doc/dnn3.html) also uses the parallel approximate Natural Gradient SGD [28] but the structure of the network changes to a Time Delay Neural Network (TDNN). TDNNs were introduced in ASR long time ago [30]. They are DNNs designed to recognize sequences of input vectors (as speech parameter vectors) so that they could be aware of time variations and at the same time provide some time shift invariance to avoid the need for a precise segmentation. The main idea is that the output of an unit at a time step depends on the outputs of the previous layer at a time interval including that time step, with different weigths for different time steps and previous layer units. In this way, the network can learn temporal patterns and at the same time the output of TDNN layers is somehow insensitive to (small) time shifts. In our case, the DNN has 6 hidden layers, and only the hidden layers 3 and 5 are TDNN layers that take as input the output of the previous layer at ±2 and ±4 frames, respectively. The non-linear activation functions of all hidden layers are *p* − *norm* as in the **FC-pnorm** system having 3000 units that are then reduced to 300 units with *p* − *norm*. The rest of the network is again similar to the **FC-pnorm**.
- **TDNN-ReLU** This network is exactly the same as the **TDNN-pnorm** DNN but changing the *p* − *norm* dimensionality reduction non-linearities by standard Rectified Linear Units (*ReLU*) non-linearities. The hidden layers have 3000 units (which are not reduced to 300 units), which results in a much larger number of parameters.

In all cases, learning rate is reduced exponentially during the first iterations and is kept fixed for a few final iterations (except for the **FC-sigmoid-RBM-pretrain**).

All the previous systems used features with a high degree of post-processing, including trained transformations and speaker adaptation. In order to analyze the influence of this post-processing and speaker adaptation we have also performed experiments with the **FC-pnorm** baseline system but using as input the raw MFCCs, Filterbank outputs, and amplitude spectrogram in dB. These will serve as a baseline to compare the systems we developed using raw multi-resolution features.

## Multi-resolution systems

We are interested in experimenting with DNNs fed with input features that include different time-frequency resolution representation of the speech analysis to verify our hypothesis that this could improve acoustic-phonetic modeling. Perhaps the easiest and most direct way to do this is by conducting different STFT analyses of the speech and combining the different spectra obtained into a single feature vector to be used as input to the DNNs. In this way, we are only modifying the input to the network, so we keep the rest of the parameters of the baseline systems described in Section Baseline systems.

**Fig 5:**
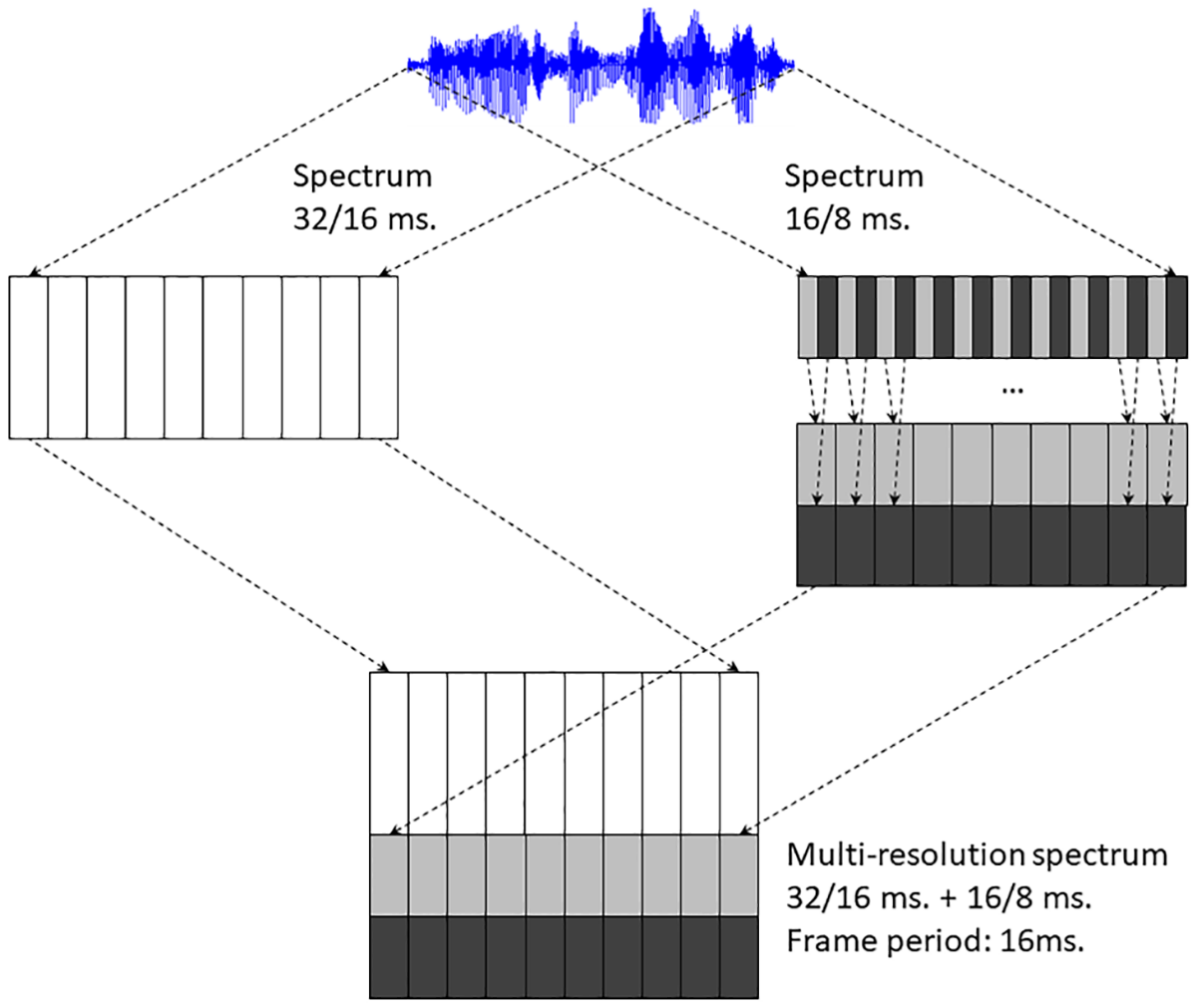
Multiresolution spectrum computation. The figure shows an example of combination of two spectra computed with different window lengths and frame periods to produce a multi-resolution spectrum with a fixed frame period.

The parameters of the STFT analysis that we modify to change the time-frequency resolution are the window length, *L*, and the frame period, *R*. In order to make it easier to use different scales we are starting our experiments with both parameters being powers of two. So we take for our baseline STFT analysis a window length of 32 ms. (*L* = 512 samples) and a frame period of 16 ms. (*R* = 256 samples). In order to include different time-frequency resolutions we compute other spectra halving both parameters each time to have analyses with window length/frame periods of: 16/8, 8/4, 4/2, 2/1, 1/0.5 and 0.5/0.25 ms. One problem that arises is that the frame period of the different STFT analyses is different, but as long as we proceed by halving the frame period this problem can be solved by re-arranging two frames of an analysis using a halved frame period as a single (double-length) vector, so that in the end the multi-resolution analysis can be represented as an augmentation of the original spectrum features at a fixed frame period of 16 ms. Fig 5 shows the scheme followed to combine two spectral analyses with different window lengths and frame periods (in this particular case 32/16 and 16/8 ms.) to form an augmented speech feature vector sequence with a fixed frame period (in this particular case 16 ms.). Using this approach recursively we can build augmented multi-resolution feature vectors representing many different time-frequency trade-offs but using a single frame period, which makes easier to use this sequence as input to a typical hybrid HMM/DNN ASR system.

In particular, we have used this approach to augment the input of the **FC-pnorm** and the **TDNN-ReLU** baseline systems using the raw spectrogram to check if this additional information helped the DNNs learn the acoustic-phonetic decoding. To double check that this was platform independent (i.e. independent of Kaldi) we also performed experiments using Fully Connected networks using Keras and Theano and evaluated the frame accuracy provided by the network.

Finally, since using the raw spectrogram provided performance below that obtained with the original features used in the baseline (which included speaker adaptation) we also used the same approach shown in Fig 5 to augment the MFCC+CMVN+Splice+LDA+MLLT+fMLLR features (used as input for all the baseline systems) so that they contain multi-resolution information, and used them as input to the best baseline system we had, the **FC-sigmoid-RBM-pretrain** system.

## Results and discussion

### Baseline results

Table 2 presents the results, in terms of Phone Error Rates (PER), obtained by the baseline systems on the TIMIT development and core test sets. All these results were obtained using features with a high degree of post-processing and including speaker adaptation. The best results are obtained with the **FC-sigmoid-RBM-pretrain** which uses unsupervised initialization of the weights of the DNN using RBMs [20]. This type of initialization is not very commonly used nowadays, particularly for large datasets, but for this rather small dataset it seems that this initialization of the network weights is still important. We should mention that this best result with the Kaldi toolkit is currently a bit far from the best published results on TIMIT, among which we have to mention a 17.7% PER using the Connectionist Temporal Classification (CTC) technique and an RNN transducer [8], a 16.7% PER using Time and Frequency Convolutional Networks [31] and a 16.27% PER using a dropout technique specifically designed for RNNs [32], all of them in the core test set. Anyway, in this work our intention is not to outperform the current state of the art systems, but to check if our proposed multi-resolution strategy can improve our baseline results.

**Table 2:** Baseline results with Kaldi. In all cases features are MFCC+CMVN+Splice+LDA+MLLT+fMLLR (the same used in the **Triphones3** HMM/GMM setup. Feature splicing indicated in the table is performed at the input of the DNN. *Input Dim*. is the dimension of the input of the network including feature splicing. *Param*. is the number of trainable parameters of the network.

DNN type and activations	Feature Splicing	Input Dim.	Param. (×10^6^)	% PER (Dev)	% PER (Test)
**FC-sigmoid-RBM-pretrain**	(±5)	440	7.78	**17.5**	**18.5**
**FC-tanh**	(±4)	360	1.14	21.1	22.6
**FC-pnorm**	(±4)	360	2.57	19.6	21.1
**TDNN-pnorm**	(±4)	360	7.25	18.7	20.6
**TDNN-ReLU**	(±4)	360	69.21	19.5	21.0

### Results with simplified features

Table 3 presents results obtained with the **FC-pnorm** setup with simplified features. The first row corresponds to the baseline **FC-pnorm** system with the fully processed and speaker-adapted features. The rest of the rows correspond to the same system with speaker-independent simplified features, and presents a clear degradation with respect to the baseline system. This is important because many of our experiments are performed on raw spectrograms and we have to take this into account to properly interpret the results. We have included a last row in which the **FC-pnorm** setup has been modified to include a third hidden layer. Given that the spectrogram amplitudes are very raw features, using them probably requires more processing levels in the DNN. Results seem to confirm this, so we have decided to use three layers in the rest of the experiments with spectrograms and the **FC-pnorm** setup.

**Table 3:** Results with simplified features. In all cases the DNN is similar to the **FC-pnorm** with the only difference that the input layer is modified to fit the input dimensionality. In all cases a frame splicing of ±4 is used at the input of the DNN. *Input Dim*. is the dimension of the input of the network including feature splicing. *Param*. is the number of trainable parameters of the network.

Feature Description	Input. Dim.	Param. (×10^6^)	% PER (Dev)	% PER (Test)
**MFCC+CMVN+Splice+** **+LDA+MLLT+fMLLR**	360	2.57	**19.6**	**21.1**
**MFCC**	117	1.84	22.2	24.3
**Filterbank**	216	2.11	24.1	26.1
**Spectrogram**	2313	8.43	22.1	24.2
**Spectrogram (3 hidden layers)**	2313	9.33	20.7	23.5

### Multi-resolution results

In this section we present our results using multi-resolution speech analysis. Our first experiment consists in using a system with the **FC-porm** setup with three hidden layers (identical to the last row in Table 3) and compare results using as input a single-resolution spectrogram and a multi-resolution spectrogram using different window lengths and frame shifts. To simplify multi-resolution analysis we use window lengths and frame shifts that are powers of two, so we start with a single-resolution system using a window length of 32 ms. and a frame shift of 16 ms. Results using this system are shown in the first row of Table 4. They are very similar to the system shown in the last row of Table 3 which uses a window length of 25 ms. and a frame shift of 10 ms. The other lines of the table show results using the same system with multi-resolution systems including 32 and 16 ms. window lengths with 16 and 8 ms. frame shifts respectively, and progressively adding a window length and a frame shift that are half of the smallest previous ones. Results show that when adding multiple resolutions, PER tends to decrease, reaching a minimum with 3 window lengths (32, 16 and 8 ms.) which is slightly better (0.6% absolute PER improvement) than using the single-resolution spectrum.

**Table 4:** Results with multi-resolution spectrograms and FC-pnorm networks. In all cases DNNs have three hidden layers and include ±4 splicing of input features features, which are raw spectrograms in dB obtained with Hamming windows. *Input Dim*. is the dimension of the input of the network including feature splicing. *Param*. is the number of trainable parameters of the network.

Multi-resolution Spectrum (window lengths)	Input Dim.	Param. (×10^6^)	% PER (Dev)	% PER (Test)
32 ms.	2313	9.35	21.3	23.4
32+16 ms.	4635	16.31	21.2	23.1
32+16+8 ms.	6975	23.33	**20.6**	**22.8**
32+16+8+4 ms.	9351	30.46	20.7	23.0

To rule out the possibility that this result was something particular to this setup we repeated essentially the same experiment with the **TDNN-ReLU** setup and present results in Table 5. These results show a similar trend: PER tends to improve as we add more time-frequency resolutions to our speech analysis. The main difference with respect to Table 4 is that this time the minimum is reached when 6 different time-frequency resolutions are used (window lengths from 32 to 1 ms. and frame shifts from 16 to 0.5 ms. in powers of two). Probably, the reason for this is that this DNN is much more complex than the **FC-pnorm** network used in Table 4 (in particular the number of parameters is about 10 times larger). This seems to allow the DNN to learn better from the very large-dimensionality input vectors. In fact, the improvement using multi-resolution speech analysis in this table (absolute reduction of 2.2% in PER from the single-resolution system in the first row) is much better than that obtained in Table 4. Actually, the 20.3% PER obtained using **TDNN-ReLU** and multi-resolution spectrum outperforms all baseline systems shown in Table 2, with the only exception of the **FC-sigmoid-RBM-pretrain** system. We should remark that this is achieved using raw multi-resolution spectrograms that are not processed in any way (except for being normalized to have zero mean and unit variance) and are not speaker-adapted as all the features used for results in Table 2 are.

**Table 5:** Results with multi-resolution spectrograms and TDNNs-ReLU networks with ±10 feature splicing. In all cases features are raw spectrograms in dB obtained with Hamming windows. *Input Dim*. is the dimension of the input of the network including feature splicing. *Param*. is the number of trainable parameters of the network.

Multi-resolution Spectrum (window lengths)	Input Dim.	Param. (×10^6^)	% PER (Dev)	% PER (Test)
32 ms.	5397	75.94	20.3	22.5
32+16 ms.	10815	82.91	19.7	21.6
32+16+8 ms.	16275	89.93	19.4	20.9
32+16+8+4 ms.	21819	90.06	19.3	20.7
32+16+8+4+2 ms.	27531	104.40	19.3	20.9
32+16+8+4+2+1 ms.	33579	112.18	**19.0**	**20.3**
32+16+8+4+2+1+0.5 ms.	40299	120.81	19.2	21.0

Previous results showed consistent improvements in terms of PER when multi-resolution speech analysis is used as input to the DNNs. However, all previous results were obtained using the Kaldi toolkit, so we performed a different experiment in which we extracted the same training examples and used Keras and Theano to train a standard Fully Connected DNN with ReLU activations in the hidden layers and *softmax* activation in the output layer to classify the input frames as one of the 1936 phone states. The optimizer used is Adam and the networks were trained for a total of 20 epochs. In Table 6 we show frame accuracies obtained with single resolution and multi-resolution spectra as input to the DNN. We can observe the same trend as in previous tables: results tend to improve as more time-frequency resolutions are considered, up to a certain point. In this case the best results are obtained at an intermediate point between the one found in Table 4 and that found in Table 5. In particular the best result is achieved with 32, 16, 8 and 4 ms. windows, reaching a frame accuracy of 38.07%, which represents an absolute improvement of 3.7% in frame accuracy from the single-resolution system in the first row.

**Table 6:** Results with multi-resolution spectrograms and Fully Connected feedforward DNNs trained with Keras and Theano with ReLU activation functions and ±4 feature splicing. In all cases features are raw spectrograms in dB obtained with Hamming windows. Results are given as frame by frame phone state recognition accuracy considering 1936 different phone states. *Input Dim*. is the dimension of the input of the network including feature splicing. *Param*. is the number of trainable parameters of the network.

Multi-resolution Spectrum (window lengths)	Input Dim.	Param. (×10^6^)	% Acc. (Dev)	% Acc. (Test)
32 ms.	2313	21.99	35.31	34.37
32+16 ms.	4635	28.89	37.23	36.51
32+16+8 ms.	6975	35.91	38.26	37.89
32+16+8+4 ms.	9351	43.04	**38.67**	**38.07**
32+16+8+4+2 ms.	11799	50.38	38.58	38.06
32+16+8+4+2+1 ms.	14391	58.16	37.95	37.33

Our previous results with multi-resolution speech analysis were consistent and encouraging. However, they start with a huge disadvantage that becomes clear observing Table 3 again. Here we see that from the speaker-adapted and highly processed features (first row of the table) to the raw spectrogram features (4^th^ row of the table) we lose over 3% absolute PER. We mitigate this a bit (last row of the table) by using more hidden layers (since we are using less processed features) but still we lose 2.4% absolute PER. So the natural question to ask is: can we use a multi-resolution approach and still get the benefits from speaker-adapted and highly processed features? We have applied a very simple approach to address this question, with the hope that it is good enough to show that it is possible. We have used modified Kaldi scripts to obtain speaker-adapted and highly processed features (MFCC+CMVN+Splice+LDA+MLLT+fMLLR) using different configurations for speech analysis. We kept the baseline speech processing, using 25 ms. windows with a frame period of 10 ms., 23 filters in the Mel-scaled filterbank and 13 MFCCs, to obtain the fully processed features corresponding to 25 ms. Then we added other fully processed features computed using other configurations, as shown in Table 7. Given that the original scripts were written using a frame period of 10 ms., we decided to use this final frame period for our multi-resolution features. For windows larger than the original (32 ms.) we kept the same frame period of 10 ms. However, for smaller windows (16, 8 and 4 ms.) we decided to use smaller frame periods (*Initial frame Period* in the table) and combine several consecutive feature vectors into a single feature vector as represented in the right part of Fig 5. Specifically, for each window length we combine exactly the number or feature vectors indicated in column *Initial frames per final frame* to have in all cases a final frame period of 10 ms. Once we have the final speaker-dependent features including MFCC+CMVN+Splice+LDA+MLLT+fMLLR for each window length we combine them to form larger input vectors using again the scheme shown in Fig 5 with the important difference that, in this case, the vectors are not spectra but these highly processed and speaker-adapted feature vectors derived from the MFCCs. We have decided to keep all the MFCCs available from the filterbank output to avoid losing information in the DCT transformation. We also decided to use a different number of filters in the filterbank for different window lengths because shorter durations of the window implied less frequency resolution and, therefore, we will require less filters for a representation with less frequency resolution (besides, for the smallest window of 4 ms., the spectrum has only 33 points computed from a 64-point DFT and it was impossible to fit a mel-scaled filterbank with 10 or more filters).

**Table 7:** Configurations used with multi-resolution highly processed and speaker-adapted features and FC-sigmoid-RBM-pretrain DNNs.

Window length (ms)	Initial frame period (ms)	# Filters in FBank	# MFCCs	# Initial frames per final frame	Final frame period (ms)
25	10	23	13	1	10
32	10	32	32	1	10
16	5	16	16	2	10
8	2.5	8	8	4	10
4	1.25	4	4	8	10

Besides the use of simplified, speaker-unadapted features, previous results shown in this paper present a second disadvantage that becomes clear observing Table 2. the best result obtained in our baseline systems uses the **FC-sigmoid-RBM-pretrain** setup which achieves 2.1% better PER in absolute terms than any of the other setups, probably due to the RBM pretraining and the small size of the dataset.

Our next experiments try to overcome these two disadvantages by combining multi-resolution highly post-processed and speaker-adapted features with the **FC-sigmoid-RBM-pretrain**. Table 8 shows the initial results we obtained. Given that the differences were very small we performed 10 different experiments with 10 different random seeds and present the mean and the standard deviation. Mean results for the baseline (first row of Table 8) are slightly different from the results obtained using the standard Kaldi random seed (first row of Table 2). Multi-resolution features achieved slight improvements (less than 0.2% absolute PER) in development and in the core test set for all the the cases except the one including the 4 ms. windows. A Wilcoxon signed rank test shows that the difference between the baseline and the best result (32+25 ms.) is statistically significant at the 0.05 level for the development results but not for the test results.

**Table 8:** Results with multi-resolution features and FC-sigmoid-RBM-pretrain DNNs with hidden layers of 1024 units. In all cases features are MFCC+CMVN+Splice+LDA+MLLT+fMLLR. Input is spliced ±5 frames. *Input Dim*. is the dimension of the input of the network including feature splicing. *Param*. is the number of trainable parameters of the network.

Multi-resolution Window lenghts	Input Dim.	Param. (×10^6^)	% PER (Dev)	% PER (Test)
25 ms. (Baseline)	440	7.78	17.52 ± 0.08	18.90 ± 0.20
32+25 ms.	880	8.13	**17.37 ± 0.14**	**18.73 ± 0.20**
32+25+16 ms.	1320	8.58	17.42 ± 0.10	18.84 ± 0.22
32+25+16+8 ms.	1760	9.04	17.46 ± 0.13	18.81 ± 0.15
32+25+16+8+4 ms.	2200	9.49	17.62 ± 0.13	18.96 ± 0.29

%PER is shown as *mean* ± *s*.*d*. obtained in 10 runs with different random seeds.

We suspected, based on the results of Tables 4 and 5, that this result was in part due to the small number of parameters of the **FC-sigmoid-RBM-pretrain** DNN, so we decided to increase the number of weights of the DNN by doubling the number of units in the hidden layers. This leads to results in Table 9, in which we can observe again that in general PER results improve with multi-resolution features (except for the cases in which the 4 ms. window is applied). The best results (obtained with windows of 25, 16 and 8 ms.) show an absolute decrease in PER of 0.64% and 0.65% in PER with respect to the baseline system of Table 8 in development and in the core test set, respectively. However a good part of this improvement is due to the increase in complexity of the DNN: the first row of Tables 8 and 9 shows that increasing the number of units in the hidden layers from 1024 to 2048 and keeping the single resolution features yields a improvement of 0.20% absolute PER in development and a 0.32% in the core test set. A Wilcoxon signed rank test finds these two differences statistically significant at the 0.05 level. The improvement achieved by the use of multi-resolution features (comparing with the first row of Table 9) is a bit larger than that found with 1024 hidden layers (Table 8), but still very small: 0.44% in development and 0.32% in the core test set. In this case, a Wilcoxon signed rank test shows that these two differences are statistically significant at the 0.05 level.

**Table 9:** Results with multi-resolution features and FC-sigmoid-RBM-pretrain DNNs with hidden layers of 2048 units. In all cases features are MFCC+Splice+LDA+fMLLR. Input is spliced (±5 frames). *Input Dim*. is the dimension of the input of the network including feature splicing. *Param*. is the number of trainable parameters of the network.

Multi-resolution Window lenghts	# Input Dim.	# Parameters (×10^6^)	% PER (Dev)	% PER (Test)
25 ms.	440	25.85	17.32 ± 0.13	18.57 ± 0.23
25+16 ms.	880	26.75	17.04 ± 0.12	18.37 ± 0.16
25+16+8 ms.	1320	27.65	**16.88 ± 0.14**	**18.25 ± 0.19**
25+16+8+4 ms.	1760	28.56	17.61 ± 0.19	19.06 ± 0.20
32 ms.	440	25.85	17.61 ± 0.19	19.15 ± 0.22
32+16 ms.	880	26.75	17.34 ± 0.13	18.47 ± 0.07
32+16+8 ms.	1320	27.65	17.04 ± 0.15	18.31 ± 0.20
32+16+8+4 ms.	1760	28.56	17.29 ± 0.14	18.70 ± 0.24
32+25 ms.	880	26.75	17.19 ± 0.13	18.53 ± 0.31
32+25+16 ms.	1320	27.65	17.19 ± 0.13	18.38 ± 0.31
32+25+16+8 ms.	1760	28.56	**16.88 ± 0.11**	18.28 ± 0.16
32+25+16+8+4 ms.	2200	29.46	17.19 ± 0.09	18.55 ± 0.21

%PER is shown as *mean*±*s*.*d*. obtained in 10 runs with different random seeds.

We have also computed the frame accuracies obtained with the DNNs used in Tables 8 and 9. Frame accuracies provide a different view of the results with more resolution (since it is evaluated frame by frame instead of phone by phone) and focusing more on the DNN itself (because no language modeling is taken into account). Frame accuracies are shown in Tables 10 and 11.

**Table 10:** Frame accuracies with multi-resolution features and FC-sigmoid-RBM-pretrain DNNs with hidden layers of 1024 units. In all cases features are MFCC+CMVN+Splice+LDA+MLLT+fMLLR. *Input Dim*. is the dimension of the input of the network including feature splicing. *Param*. is the number of trainable parameters of the network.

Multi-resolution Window lenghts	Input Dim.	Param. (×10^6^)	% Acc. (Dev)	% Acc. (Test)
25 ms. (Baseline)	440	7.78	**52.12 ± 0.10**	**51.34 ± 0.15**
32+25 ms.	880	8.13	51.98 ± 0.11	51.07 ± 0.13
32+25+16 ms.	1320	8.58	51.80 ± 0.16	51.02 ± 0.16
32+25+16+8 ms.	1760	9.04	51.73 ± 0.12	50.98 ± 0.16
32+25+16+8+4 ms.	2200	9.49	51.17 ± 0.09	50.36 ± 0.15

%Acc. is shown as *mean*±*s*.*d*. obtained in 10 runs with different random seeds.

**Table 11:** Frame accuracies with multi-resolution features and FC-sigmoid-RBM-pretrain DNNs with hidden layers of 2048 units. In all cases features are MFCC+Splice+LDA+fMLLR. *Input Dim*. is the dimension of the input of the network including feature splicing. *Param*. is the number of trainable parameters of the network.

Multi-resolution Window lenghts	# Input Dim.	# Parameters (×10^6^)	% PER (Dev)	% PER (Test)
25 ms.	440	25.85	52.66 ± 0.12	51.95 ± 0.17
25+16 ms.	880	26.75	53.12 ± 0.08	52.53 ± 0.15
25+16+8 ms.	1320	27.65	**53.16 ± 0.08**	**52.63 ± 0.13**
25+16+8+4 ms.	1760	28.56	51.04 ± 0.13	50.63 ± 0.18
32 ms.	440	25.85	51.52 ± 0.11	52.17 ± 0.17
32+16 ms.	880	26.75	52.70 ± 0.06	52.05 ± 0.10
32+16+8 ms.	1320	27.65	52.93 ± 0.13	52.31 ± 0.11
32+16+8+4 ms.	1760	28.56	52.18 ± 0.09	51.47 ± 0.08
32+25 ms.	880	26.75	52.99 ± 0.08	52.17 ± 0.13
32+25+16 ms.	1320	27.65	52.90 ± 0.13	52.16 ± 0.15
32+25+16+8 ms.	1760	28.56	53.02 ± 0.10	52.20 ± 0.10
32+25+16+8+4 ms.	2200	29.46	52.32 ± 0.12	51.70 ± 0.12

%PER is shown as *mean*±*s*.*d*. obtained in 10 runs with different random seeds.

Results with 1024 units in the hidden layers (Table 10) are a bit surprising because no improvement is found in frame accuracy when applying multi-resolution features, while small improvements were found in PER (with the only exception of the combination including windows of 4 ms.). A possible explanation for this could be that in this case multi-resolution may be helping the DNN to make softer estimations of the frame-by-frame posterior probabilities, so that even being less accurate frame by frame results are slightly better when integrated over phone durations. In any case, the differences found in PER and frame accuracy with 1024 hidden layers are very small.

Results with 2048 units in the hidden layers (Table 11), however, follow a similar trend to results in PER with 2048 units in the hidden layer (Table 9). We can observe again that frame accuracy tends to be better when multi-resolution features are applied. The best results are obtained again for the same combination of windows (25, 16 and 8 ms.) and represent an absolute improvement of 0.5% and 0.68% in frame accuracy over the result of the first row with single-resolution features. These differences in frame accuracies are clearly statistically significant (a Wilcoxon signed rank test confirms that they are statistically significant even at the 0.01 level).

## Conclusion

Normally, automatic speech recognition starts with a Short-Time Fourier Transform (STFT) which defines a fixed point in the time-frequency resolution trade-off. This approach, traditionally followed by the calculation of Mel-Frequency Cepstral Coefficients (MFCC) that produced reasonably uncorrelated features was very well suited to the *old* state-of-the-art in Automatic Speech Recognition (ASR), dominated by the use of Hidden Markov Models (HMMs) to model the speech dynamics and Gaussian Mixture Models (GMMs) (with diagonal covariance matrices) to model the features extracted from a speech frame. Nowadays, one of the most commonly used frameworks in practical ASR systems consists on the adoption of Deep Neural Networks (DNN) as a replacement of the GMMs, giving rise to the *hybrid* HMM/DNN systems. For these systems, since the acoustic features are fed directly as the input to a DNN, several restrictions on the speech features have vanished. For instance, input features do not need to be uncorrelated, and we have more freedom to enlarge the input feature vector because DNNs can handle better the *curse of dimensionality*.

In this context, and knowing that speech phones vary considerably in length (from an average of less than 18 ms. for the /*b*/ phone to over 150 ms. for the /*aw*/ phone in the TIMIT corpus) we have hypothesized that using multiple time-frequency resolution trade-offs would be beneficial to model the diversity in time-frequency resolutions present in the speech signal.

To confirm our hypothesis we have experimented taking as baseline the Kaldi TIMIT recipes and extending them to have a complete set of baseline *hybrid* HMM/DNN systems. We have modified these systems because they worked on highly post-processed and speaker-adapted features (MFCC+CMVN+Splice+LDA+MLLT+fMLLR) and we were interested to test our hypothesis directly on spectral features. Our experiments showed that including multi-resolution spectral features (by concatenating features with different time-frequency resolutions as the input vector) provided consistent improvements using different setups for the DNNs (this included a **FC-pnorm**, a **TDNN-ReLU** using Kaldi and a conventional Fully Connected DNN built using Theano and Keras). While our results are consistent in showing that multi-resolution speech analysis improves Phone Error Rate (PER) and frame classification accuracy, we obtained differences in terms of the amount of multi-resolution that provided the best results and we hypothesized that this difference was due to the different complexities of the DNNs, with more complex (in terms of the parameters) DNNs having more capability to exploit multi-resolution information.

Results using raw multi-resolution spectral features were able to provide slightly better results than equivalent systems using highly post-processed and speaker-adapted features. However, using raw spectral features instead of highly post-processed and speaker adapted features, as well as not using the best Kaldi DNN setup for TIMIT (**FC-sigmoid-RBM-pretrain**) as a starting point, was a handicap to improving the best baseline results. In our last experiments we used this best Kaldi DNN setup for TIMIT as starting point, but replaced the original features with multi-resolution features including all the post-processing and speaker adaptation of the baseline Kaldi systems (MFCC + CMVN + Splice + LDA + MLLT + fMLLR). Combining this with an increase in the complexity of the DNN (changing the number of units in the hidden layers from 1024 to 2048) we achieved an improvement with respect to the best results provided by Kaldi, which are lowered from 18.90% to 18.25% PER on TIMIT core-test set. These results are the mean of 10 runs with different random seeds (a single run with the standard Kaldi seed provided an improvement from 18.5% to 17.9%). A deeper analysis of this result showed that just by changing the number of units in the hidden layers from 1024 to 2048 improved PER from 18.90% to 18.57%, while multi-resolution provided the remaining improvement from 18.57% to 18.25%, which is a very limited improvement but resulted in a statistically significant difference. We analyzed the frame accuracies obtained with similar results: we improved from 51.34% to 52.63% combining an increase in the number of hidden units and multi-resolution, but just the use of multi-resolution provided an improvement from 51.95% to 52.63%, which again is small but statistically significant.

Our results are far from the best published results on TIMIT (16.27% PER in [32]), but our goal in this article was not to beat the state of the art, but to check whether a multi-resolution speech representation helped in DNN based acoustic modeling for ASR. Our experiments so far seem to confirm that a multi-resolution speech representation tends to improve ASR results of *hybrid* HMM/DNN systems. However, our experiments also seem to indicate that multi-resolution can provide larger improvements when raw spectral features are used, providing only very small improvements when highly processed and speaker-adapted features are used (or perhaps when results are already very good). Moreover, to obtain these small improvements it seems to be necessary to increase the complexity of the DNNs to deal with the additional input information included in the multi-resolution features (our experiments seem to indicate that using multi-resolution with a DNN optimized for single resolution yield only very small improvements). Besides, all of the experiments presented in this work have been restricted to a single corpus of read and clean speech, TIMIT, which is also very limited in size. Therefore, it is definitely necessary to deepen our research in the future to better understand the role of multi-resolution in ASR, and to propose better ways to explode this information. Part of this future research should be to extend our experiments to other corpora including more data with different features (in particular including spontaneous speech) to confirm if our findings are generally applicable. One of the most interesting conclusions of our results is that they seem to indicate that ASR results could potentially be improved by applying this multi-resolution strategy in the feature extraction part of the ASR system, and therefore this improvement could potentially be applied to any existing ASR system using DNNs. In particular, given that multi-resolution improvements with already good performing systems seem to be very limited, it would be very interesting to apply multi-resolution to the best performing systems on TIMIT such as [32] to check if multi-resolution can improve the most accurate systems on TIMIT.
